# Yield and Efficiency of Mental Disorder Screening at Intake to Prison: A Comparison of DIA-X Short- and Long-Screening-Protocols in Compensation Prisoners

**DOI:** 10.3389/fpsyt.2018.00538

**Published:** 2018-10-26

**Authors:** Carola Schildbach, Sebastian Schildbach

**Affiliations:** Praxisgemeinschaft für Psychosomatik, Gais, Switzerland

**Keywords:** compensation imprisonment, mental disorder screening, DIA-X, sensitivity and specificity, predictive values

## Abstract

**Background:** Inmates are several times more likely to suffer from mental disorders than the general population.In order to take appropriate curative or preventive measures, a precise psychiatric diagnosis at detention start would therefore be imperative, but is frequently not carried out for reasons of time. The computer-aided expert system DIA-X enables a rapid and reliable diagnosis of psychiatric disorders. DIA-X is available as a short screening questionnaire with a processing time of a few minutes and as a standardized interview, which takes ~1 h to complete.

**Objective:** The aim of this study was to assess the efficiency and accuracy of the DIA-X short screening questionnaire.

**Methods:** One hundred detainees were recruited randomly from compensation prisoners, who were imprisoned because they were unwilling or unable to pay a fine for committing a criminal offence, from the penal institution Berlin-Plötzensee in 2017. Both the short screening questionnaire and the standardized interview from the DIA-X expert system were used for diagnosing mental disorders. Based on the results of the standardized interview from four study populations of compensation prisoners from 1999, 2004, 2010, and 2017, the sensitivity, specificity and the predictive values of the screening form were inferred.

**Results:** More than half of the compensation prisoners suffered from mental and behavioral disorders caused by the abuse of alcohol or psychoactive substances. Phobic anxiety disorders were detected in one out of ten compensation prisoners and two out of ten compensation prisoners suffered from major depressive disorders. The DIA-X screening questionnaire was able to detect all mental illnesses with a sensitivity of 100%. However, specificities were low for nicotine dependency, drug and alcohol abuse. High specificities and high predictive values were obtained for psychoses and anxiety disorders.

**Conclusions:** As the main test quality criteria of the DIA-X screening forms were so low, we cannot recommend the application of the DIA-X screening form for obtaining a valid diagnosis. Therefore, we explicitly recommend using the long form DIA-X for the detection of the most serious cases of mental illness. Then, these prisoners could receive either therapy or special social training.

## Introduction

At present, there are between 9 and 10 million people in prisons worldwide (1), and an even larger number of former prisoners live in society (2). Therefore, determining the physical and mental health status of current and former inmates is an important issue in public health.

In Germany, approximately 80% of all penalties are imposed as monetary fines. If a convict is unwilling or unable to pay the fine for committing a criminal offense, he or she can face compensation imprisonment instead, as regulated under Section 43 of the German Penal Code (3, 4).

Thus, compensation imprisonment ensures the effectiveness of the penalty system. Despite small variations between the different federal provinces, the proportion of compensation prisoners amounts for ~10% of all inmates in Germany (5).

The duration of the compensation imprisonment corresponds to the number of daily rates that an offender was sentenced to pay. The number of daily rates corresponds to the severity of the crime, and if for example the court sanctioned a fine of 30 daily rates, then the convicted person faces a 30-day compensation imprisonment. At the same time, a compensation imprisonment can be averted by paying the fine or by completing community service (4).

However, the application of compensation imprisonment is subject to an ongoing discussion in Germany (6–8). As the average stay in prison is short, there are no decisive concepts for social rehabilitation after imprisonment. In addition to a lack of resocialization, potential job loss and social stigmatization, the newly acquired subcultural contacts facilitate reoffending (9).

Numerous epidemiologic studies demonstrated that prisoners are more likely to suffer from mental disorders than the average population (1, 10–12). In addition to the observation that the majority of prison inmates (81%) were male, 3.7% of male, and 4% of female inmates experienced psychotic disorders, 10% of males and 12% of female inmates showed signs of depression and 65% of the male and 42% of female inmates were diagnosed with personality disorders (1).

In comparison to the American or British average population of the same age (13, 14), prisoners suffered from psychotic illnesses, severe depression and dissocial personality disorders 2–10 times more frequently (1, 15, 16).

As compensation imprisonment only exists in Germany, Austria and Switzerland, there are only a few representative medical studies on the prevalence of mental disorders of compensation prisoners. Four consecutive studies from 1999, 2004, 2010, and 2017, each consisting of 100 prisoners of the penal institution Berlin-Plötzensee, found a high rate of mental and behavioral disorders in compensation prisoners, mainly due to the abuse of alcohol and drugs (17–19).

In order to support the detainees during their detention and after their release to reintegrate into society, it is necessary to make a precise diagnosis of pre-existing mental illnesses. To this end, computer-assisted diagnostic systems are available for a standardized, independent, and reliable diagnosis. Unfortunately, a precise psychiatric diagnosis at detention start is often not carried out for reasons of time.The frequently used psycho-diagnostic system DIA-X exists as a long version that takes an hour to answer, and as a short version that can be answered within a few minutes.

The aim of this study was to assess the sensitivity and specificity of the short version of DIA-X using the results of the long version of DIA-X in a study population of 100 compensation prisoners.

## Study population and methods

### Study population

The study population consisted of 100 randomly selected inmates of the penal institution Plötzensee in Berlin, who served compensation imprisonment in spring 2017. The only inclusion criterion was a good knowledge of the German language. All study participants gave their informed consent to participate in this study.

Table 1 shows the sociodemographic characteristics of the study population. The inmates were exclusively male, on average 37.2 years old, mostly single and unemployed. Half of the inmates were convicted of fare evasion. The average number of daily rates was 106. The average penalty fee was1659 €. Thirty-eight inmates said they did not have a permanent home, and 41 inmates did not have any vocational training.

**Table 1:** Demographic characteristics of the study population in 2017 (*n* = 100).

**Demographic parameter**	**Frequency**
Mean age in years	37.2
Proportion of singles	92%
Proportion of inmates with no fixed address or in residential facilities	38%
Inmates without a school-leaving qualification	30%
Inmates without a vocational training	41%
Unemployment rate	85%
Convicted for fare evasion	49%
Convicted for other petty crimes	15%
Convicted for property crimes	21%
Convicted for personal injuries	7%
Convicted for road traffic offences	5%
Convicted for insulting others	3%
Nicotine dependency	58%
Cannabis abuse	33%
Opioid abuse	30%
Cocaine abuse	21%
Other stimulant abuse	20%
Hallucinogen abuse	12%
Sedative, hypnotic or anxiolytic-related abuse	6%

### Study approval

In January 2016, a comprehensive research proposal was submitted to the criminal services of the penal institutions in Berlin and to the social services of the penal institution Plötzensee, which were both approved in February 2016. In addition, the prison management of the penal institution Plötzensee approved the study in April 2016. Finally, the Berlin Commissioner for Data Protection issued a clearance certificate in May 2016.

### Diagnostic system DIA-X

For diagnosing psychiatric disorders, the computer-aided expert system DIA-X was used (20). DIA-X supports the user reliably and efficiently with the diagnosis according to ICD-10 (International Classification of Diseases) and DSM-IV (Diagnostic and Statistical Manual of Mental Disorders) (21).

For this investigation, we used to different versions of DIA-X:

1. A **screening procedure**, which captures fear, depression or mental disorders in general. The screening procedures are short questionnaires that either confirm or deny with high sensitivity and good specificity either the presence of any mental disorder (DIA-SSQ), anxiety disorder (DIA-ASQ), or depression (DIA-DSQ). If the suspicion of a mental disorder is confirmed, the structured interview should be used for further clarification. In addition, the screening questionnaires can also be used to measure change. The DIA-SSQ questionnaire has 17 questions, the other two 15 questions each. Each question is binary, i.e., it can only be answered with yes or no.
2. A **standardized interview** for measuring mental disorders in the last 12 months. The interview is available in two different versions: one to record the longitudinal symptoms (over the entire lifetime), the other centered on the cross-sectional symptoms (the last 12 months). Both versions are fully standardized and provide diagnoses of about 100 mental disorders according to ICD-10 and DSM IV. The modular structure and the possibilities of branching ensure that despite the standardization only the symptom constellation important for the respective subject is placed in the center of the interview becomes. In addition, some complexes can be selected. There is a supplementary booklet to the actual interview booklet, in which the examined person gives information on the symptoms, which are deepened in the interview. The information in the supplement also serves as a reminder.

Both variants of DIA-X were applied as computer versions. While it usually takes no more than 2 min to answer the screening questionnaires, the standardized interview takes about an hour to complete. In addition to the DIA-X components, the detainees were also asked questions of demographic nature.

The interviews were conducted by a general practitioner (in 1999), a criminologist (in 2004), a psychologist (in 2010), and by a social pedagogue (in 2017) (19).

### Statistical data analysis

The recorded data were instantly anonymized and encoded. The prison did not receive any information whatsoever concerning data related to individual prisoners.

By comparing the two DIA-X versions—the short screening questionnaire vs. the long detailed standardized interview—the sensitivity and specificity of the DIA-X screening questionnaire were calculated for the individual diagnoses. The diagnoses obtained with the standardized interview were considered as gold standard.

The sensitivity of the screening questionnaire for a particular mental illness was the proportion of inmates, which were tested positive for this particular mental illness and that really suffered from that particular mental illness, of all the inmates that were actually diagnosed with a particular mental disorder. $Sensitivity=\;\frac{TP}{TP+FN}$, where TP, true positive; FN, false negative.

The specificity of the screening questionnaire for a particular mental illness was the proportion of inmates, which were tested negative for this particular mental illness and that really did not suffer from that particular mental illness, of all the inmates that were actually free of a particular mental disorder. $Specificity=\;\frac{TN}{TN+FP}$, where TP, true negative; FN, false positive.

Including the prevalence of diverse mental disorders in the study population as assessed with the long form of DIA-X, the positive and negative predictive values were calculated. To this end, the average prevalence of each mental illness was calculated from the frequencies of mental disorders in four study populations of 100 compensation prisoners each collected in the penal institution Plötzensee in 1999, 2004, 2010, and 2017 (19).

The positive predictive value (PPV) is the probability that detainees with a positive DIA-X screening test truly have the specific mental disease.

$$\begin{array}{l} PPV=\frac{Sensitivity\cdot Prevalence}{\left\{Sensitivity\cdot Prevalence+\left(1-Specificity\right)\cdot \left(1-Prevalence\right)\right\}} \end{array}$$

The negative predictive value (NPV) is the probability that detainees with a negative DIA-X screening test truly don't have the disease.

$$\begin{array}{l} NPV=\;\frac{Specificity\cdot \left(1-Prevalence\right)}{\left\{\left[Specificity\cdot \left(1-Prevalence\right)\right]+\left[\left(1-Sensitivity\right)\cdot Prevalence\right]\right\}} \end{array}$$

## Results

### Mental disorders

Table 2 summarizes the average prevalence of mental disorders in four study populations, each consisting of 100 compensation imprisoners, from the years 1999, 2004, 2010, and 2017. Nearly half (45.5%) of the study population suffered from mental and behavioral disorders caused by the use of various drugs (Table 2). In fact, abuse of various psychotropic substances was detected in a large proportion of inmates (Table 1).

**Table 2:** Overview of the average prevalence of mental disorders in four study populations of compensation imprisoners from the years 1999, 2004, 2010, and 2017 (*n* = 400). Multiple answers with respect to mental disorders were possible.

**ICD-10**	**Diagnosis**	**Prevalence (%)**
F10	Mental and behavioral disorders due to use of alcohol	72.75
F11, F12, F14–16	Mental and behavioral disorders due to use of opioids, cannabinoids, cocaine, or hallucinogens	45.5
F13	Mental and behavioral disorders due to use of sedatives or hypnotics	5.25
F17.2	Nicotine dependency	65.75
F20–F29	Schizophrenia, schizotypal, delusional, and other non-mood psychotic disorders	3.75
F30	Hypomania	3
F32–F33	Depressive disorders	26.25
F34.1	Dysthymia	11.5
F40	Phobic anxiety disorders	35
F41	Other anxiety disorders	5.75
F43	Reaction to severe stress, and adjustment disorders	7
F45	Somatoform disorders	16
F50	Eating disorders	2.25

Nearly three-quarters (72.75%) of the inmates had mental health problems and behavioral problems initiated by alcohol abuse. Every third detainee who served compensation imprisonment suffered from phobic disturbances. In addition, depressive, somatoform, delusional and bipolar affective disorders as well as eating disorders and dysthymia were frequent diagnoses.

### Sensitivity and specificity of the DIA-X short form

Table 3 indicates the sensitivities and specificities of the DIA-X screening questionnaire as compared to the long form (interview form) of this diagnostic system. All mental disorders were detected with a sensitivity of 100%, i.e., all inmates suffering from a particular mental disorder were correctly classified as ill.

**Table 3:** Sensitivity and specificity of the DIA-X short form as obtained by comparing its diagnostic results to those received from the DIA-X long form (*n* = 100).

**ICD-10**	**Diagnosis**	**Sensitivity (%)**	**Specificity (%)**
F10	Mental and behavioral disorders due to use of alcohol	100	50
F11, F12, F14–16	Mental and behavioral disorders due to use of opioids, cannabinoids, cocaine, or hallucinogens	100	26
F13	Mental and behavioral disorders due to use of sedatives or hypnotics	100	81
F17.2	Nicotine dependency	100	23
F20–F29	Schizophrenia, schizotypal, delusional, and other non-mood psychotic disorders	100	99
F30	Hypomania	100	76
F32–F33	Depressive disorders	100	56
F34.1	Dysthymia	100	69
F40	Phobic anxiety disorders	100	91
F41	Other anxiety disorders	100	87
F43	Reaction to severe stress, and adjustment disorders	100	82
F45	Somatoform disorders	100	96
F50	Eating disorders	100	95

Low specificities were noted for nicotine addiction (23%), drug abuse (26%), and alcohol abuse (50%). However, since nicotine addiction as well as drug abuse and alcohol abuse occurred with high prevalence in compensation prisoners (Tables 1, 2), the respective scales of the DIA-X screening method tended to overestimate.

High specificities (above 90%) were achieved for psychotic disorders, somatoform disorders, phobic anxiety disorders, and eating disorders. For these mental disorders, the ability to actually categorize healthy inmates as healthy was high.

Moderate specificities (between 56 and 87%) were achieved for hypomania, other anxiety disorders, post-traumatic disorders, mental and behavioral disorders due to the use of sedatives or hypnotics, dysthymia, and depression.

### Negative and positive predictive values

Table 4 shows the negative predictive values and the positive predictive values of the short form of DIA-X depending on the prevalence of the diagnosed mental illnesses. The positive predictive values varied between 60 and 80%, indicating that only 60–80% of inmates that were diagnosed with a specific mental disorder indeed suffered from this mental illness.

**Table 4:** Negative predictive value (NPV) and positive predictive value (PPV) of the DIA-X short form, based on the average prevalence of the respective mental disorder in compensation prisoners (*n* = 100).

**ICD-10**	**Diagnosis**	**NPV (%)**	**PPV (%)**
F10	Mental and behavioral disorders due to use of alcohol	33.2	67.4
F11, F12, F14–16	Mental and behavioral disorders due to use of opioids, cannabinoids, cocaine, or hallucinogens	20.4	80.4
F13	Mental and behavioral disorders due to use of sedatives or hypnotics	39.8	60.7
F17.2	Nicotine dependency	18.6	82.2
F20–F29	Schizophrenia, schizotypal, delusional, and other non-mood psychotic disorders	42.3	58.2
F30	Hypomania	33.9	66.7
F32–F33	Depressive disorders	35.2	65.4
F34.1	Dysthymia	38.9	61.7
F40	Phobic anxiety disorders	47.2	53.4
F41	Other anxiety disorders	42.1	58.5
F43	Reaction to severe stress, and adjustment disorders	41.5	59.0
F45	Somatoform disorders	47.6	52.9
F50	Eating disorders	34.8	65.7

For nicotine addiction and drug dependence, the negative predictive values were ~20%. Thus, only 20% of those inmates who, according to the short form of DIA-X, had neither dependency, were actually free of these addictions.

## Discussion

### Mental disorders

In this particular study population of compensation prisoners, the prevalence of mental disorders was well above that of the average population.

The average lifetime prevalence of alcohol-related mental illnesses in compensation prisoners ranges around 75% (18, 19), while Germany's general population shows a lifetime prevalence of 3–5% (22–26). For schizophrenia, the estimated lifetime prevalence for compensation prisoners was 4% (18), while in the German general population a lifetime prevalence of only 1.25% was observed (27). Similarly, dysthymia has been observed much more frequently in compensation prisoners (17, 18) than in the general population (28). The lifetime prevalence of bipolar disorder in the general population was also 0.5–5.0% (29), which was significantly lower than that of compensation prisoners (17–19). Only in the prevalence of depression did the compensation prisoners lie in the population average (28).

Apart from the work mentioned above (17–19), there is only a very sparse international data on mental illness among compensation prisoners due to the special legal situation in Germany, which otherwise exists only in Austria and Switzerland.

In the light of these results, it can be argued that compensation imprisonment is a punishment of the poor and mentally ill. Instead of enforcing the law, it rather deteriorates the situation of people at the edge of society. Therefore, a precise diagnosis of mental disorders at detention start could offer the possibility to treat the detainees as patients, and not just as criminals. With an appropriate treatment, the recurrence rate could be lowered and compensation imprisonment would indeed have an effect: a curative one, not an educative one.

### Sensitivity and specificity of the DIA-X short form

For the DIA-X screening questionnaire, a sensitivity of 86% was reported for screening for mental disorders, a sensitivity of 95% for screening for depression and a sensitivity of 96% for screening for anxiety disorders (20). In our study population, sensitivities for all ascertainable mental disorders were 100%.

For the DIA-X screening questionnaire, a specificity of 75% for screening for mental disorders, a specificity of 84% for screening for depression and a specificity of 82% for screening for anxiety disorders were published (20). In our study population, the specificities were much lower than the reported values.

### Negative and positive predictive values

For a physician performing a diagnostic test on a particular patient clientele, the sensitivity, and specificity of the test are less of a concern than the negative and positive predictive values, which are influenced by the prevalence of the disorder in a particular patient clientele. Since in detainees serving a compensation imprisonment, the prevalence of mental illnesses was significantly higher than that of the general population, the determination of the positive and negative predictive values was of great interest.

For nicotine addiction and drug dependence, the negative predictive values were ~20%. Thus, only 20% of inmates in who, according to the short form of the DIA-X, neither dependency was present, were indeed free from these addictions. This value was surprisingly low at first glance, as the prevalence of addiction in the study population was very high. However, the short forms of DIA-X were not explicitly designed for the detection of these diseases, so that the low discrimination power of the short form of DIA-X is not surprising.

For depressive episodes, dysthymia, hypochondria, alcohol disorders, somatoform disorders, specific phobias, drug abuse, or dependence and social phobia, the negative predictive values were ~30%. Thus, only 30% of inmates who, according to the short form of DIA-X, did not have any of the mental illnesses listed were actually healthy with respect to these conditions. The low discrimination power for depression is alarming, as this serious disease involves numerous compensation prisoners, which would not be detected correctly with the screening version of DIA-X.

### Limitations

This study suffers from several limitations. First, we used the full version of the diagnostic system DIA-X as gold standard. However, the gold standard should be a diagnostic procedure, which in the given case represents the most proven and best solution. For psychiatric diagnosis, the gold standard is a consensus diagnosis involving all therapists, all available sources of information and interaction observations, as well as multiple interviews. Therefore, the use of a single measurement as gold standard can certainly be regarded a limitation. However, the long interview version of DIA-X has been used in many other studies assessing mental disorders (30–32) and it has been applied as validity criterion and even gold standard for evaluating newly developed diagnostic tools for mental health (33, 34).

In view of the numbers obtained for specificities, negative and positive predictive values, we conclude that the short version of DIA-X cannot be recommended for obtaining a quick and reliable diagnosis in compensation prisoners. Experienced diagnosticians would probably also immediately recognize those persons, who were diagnosed with DIA-X as mentally ill, because of their striking psychosis-related behavior.

## Conclusions

The DIA-X's screening form proved to be highly reliable in correctly diagnosing psychosis and somatoform disorders in the special population of compensation prisoners. For depressive disorders, the specificity was 56%, so we have to assume that with regard to depression many patients would not be correctly diagnosed. Also for addictions, the predictive values were in a low range.

The main idea of our project was to evaluate the applicability of a simple and fast diagnostic screening tool for obtaining a rough, but reliable diagnosis of mental disorders in compensation prisoners. However, the main test quality criteria were so low, that we cannot recommend the application of the DIA-X screening form for obtaining a valid diagnosis.

However, as the brief imprisonment promotes social stigmatization and further threatens the basis of existence of this particular clientele, compensation prisoners need support to integrate well into society after detention. To this end, the use of the long form DIA-X would lead to the detection of the most serious cases of mental illness. Then, these prisoners could receive either therapy or special social training.

Based on the results of the epidemiological studies, which showed an extremely high prevalence of mental illnesses in compensation prisoners, and with respect to the low discrimination power of DIA-X screening form, we recommend a regular application of the DIA- X interview version in compensation prisoners. Although the use of the interview form of the DIA-X is time-consuming, it seems obligatory both for ethical reasons as well as for security reasons, since the society has a vital interest in a successful integration of compensation prisoners into a functioning social system.

## Author contributions

CS conducted the literature search, collected and evaluated the data, performed the statistical analyses and wrote the article. SS conceived and prepared the study, wrote the research proposal, communicated with the authorities, recruited the patients, organized the data collection and supported CS with statistical analyses and writing the article.

### Conflict of interest statement

The authors declare that the research was conducted in the absence of any commercial or financial relationships that could be construed as a potential conflict of interest.
